# Nesprin-2 is a novel scaffold protein for telethonin and FHL-2 in the cardiomyocyte sarcomere

**DOI:** 10.1016/j.jbc.2024.107254

**Published:** 2024-04-02

**Authors:** Chen Li, Derek T. Warren, Can Zhou, Shanelle De Silva, Darren G.S. Wilson, Mitla Garcia-Maya, Matthew A. Wheeler, Peter Meinke, Greta Sawyer, Elisabeth Ehler, Manfred Wehnert, Li Rao, Qiuping Zhang, Catherine M. Shanahan

**Affiliations:** 1 King's College London British Heart Foundation Centre of Research Excellence, School of Cardiovascular and Metabolic Medicine & Sciences, London, UK; 2 Department of Cardiology, West China Hospital of Sichuan University, Chengdu, China; 3 School of Pharmacy, University of East Anglia, Norwich, UK; 4 Randall Centre for Cell and Molecular Biophysics, School of Basic and Medical Biosciences, King's College London, London, UK; 5 Department of Cardiac Development and Remodeling, Max-Planck-Institute for Heart and Lung Research, Bad Nauheim, Germany; 6 Friedrich-Baur-Institute at the Department of Neurology, LMU University Hospital, Munich, Germany; 7 Institute of Human Genetics, University of Greifswald, Greifswald, Germany

**Keywords:** nesprin-2, telethonin, FHL-2, interaction, sarcomere organization, cardiomyopathies

## Abstract

Nesprins comprise a family of multi-isomeric scaffolding proteins, forming the linker of nucleoskeleton-and-cytoskeleton complex with lamin A/C, emerin and SUN1/2 at the nuclear envelope. Mutations in nesprin-1/-2 are associated with Emery-Dreifuss muscular dystrophy (EDMD) with conduction defects and dilated cardiomyopathy (DCM). We have previously observed sarcomeric staining of nesprin-1/-2 in cardiac and skeletal muscle, but nesprin function in this compartment remains unknown. In this study, we show that specific nesprin-2 isoforms are highly expressed in cardiac muscle and localize to the Z-disc and I band of the sarcomere. Expression of GFP-tagged nesprin-2 giant spectrin repeats 52 to 53, localized to the sarcomere of neonatal rat cardiomyocytes. Yeast two-hybrid screening of a cardiac muscle cDNA library identified telethonin and four-and-half LIM domain (FHL)-2 as potential nesprin-2 binding partners. GST pull-down and immunoprecipitation confirmed the individual interactions between nesprin-2/telethonin and nesprin-2/FHL-2, and showed that nesprin-2 and telethonin binding was dependent on telethonin phosphorylation status. Importantly, the interactions between these binding partners were impaired by mutations in nesprin-2, telethonin, and FHL-2 identified in EDMD with DCM and hypertrophic cardiomyopathy patients. These data suggest that nesprin-2 is a novel sarcomeric scaffold protein that may potentially participate in the maintenance and/or regulation of sarcomeric organization and function.

Nesprins are a family of multi-isomeric spectrin repeat (SR) containing proteins, which were initially identified as important nuclear envelope (NE) components (1). The full-length nesprin-1 and -2 giant proteins are composed of a central rod domain comprised of multiple SRs mediating protein-protein interactions, a C-terminal Klarsicht/ANC-1/Syne homology (KASH) transmembrane domain mediating NE targeting, and N-terminal calponin homology (CH) domains that bind to the actin cytoskeleton (2). Both giant isoforms are located at the outer nuclear membrane (ONM), binding to the SUN(Sad1p/UNC84)-domain-containing proteins (SUN1 and SUN2) across the luminal space, to form the linker of nucleoskeleton and cytoskeleton (LINC) bridging complex, that links the inner nuclear membrane (INM) and nuclear lamina to the ONM and actin cytoskeleton. This complex is not only crucial for nuclear morphology but also essential for force transmission and mechanotransduction between the cytoskeleton and nucleus (3). Smaller nesprin isoforms, especially nesprin-1α_2_ and 2α_1_, 2ε_2_, are highly expressed in heart and/or skeletal muscle and located at both the INM and ONM, where they interact with lamin A/C and emerin (at the INM) as well as the microtubule motor protein - kinesin light chain (KLC-1/2) at the ONM (4). Like emerin, lamin A/C and SUN1/2 proteins, mutations in either nesprin-1 or -2 are causative in autosomal dominant Emery–Dreifuss muscular dystrophy (EDMD) with heart conduction defects (CD) and dilated cardiomyopathy (DCM) (2).

In addition to the well-characterized NE roles of nesprins, isoforms lacking the NE targeting KASH domain or the CH domains are expressed *via* alternative transcription and splicing. These nesprin isoforms are proposed to be scaffold proteins in multiple subcellular compartments (5, 6, 7, 8, 9, 10). In *Drosophila* nesprin-1/Msp300 isoforms have been shown to localize at Z-discs in sarcomeres, contributing to muscle function (6, 11, 12). In mammalian cells, nesprin-2 KASH-less variants are critical for ERK1/2 and β-catenin compartmentalization in the nucleus, while the nesprin-1 p50 isoform acts to attach RNA processing bodies to MTs (7, 10). Our knowledge of the roles of these KASH-less isoforms remains extremely limited. However, we have previously shown that nesprin-1 localizes to the Z-disc of the sarcomere while nesprin-2 is present at the Z-disc, A/I junction, and the sarcoplasmic reticulum in adult skeletal muscle (13). Electron microscopy has also shown that nesprin-1 is present at the Z-disc of the sarcomere in adult mouse heart tissue (14). Furthermore, *in vitro* differentiation of mouse C2C12 myoblasts demonstrated that nesprin-1 and -2 redistributed from the NE and nucleus in myoblasts to the sarcomere in myotubes, and myotube formation was dramatically reduced following nesprin-1 depletion (1). Previous evidence also showed that there was an increase in nesprin-1, -1α_2_ and -giant during early myogenesis *in vitro* while nesprin-2 partly replaced nesprin-1 at the NE. In addition, smaller nesprin isoforms became dominant during the transition from immature to mature muscle fibers *in vivo*, suggesting complex isoform switching of nesprin-1 and -2 during muscle development (15). Taken together these data highlight the importance of nesprin isoforms in muscle cell differentiation and myogenesis and suggest that KASH-less nesprin isoforms may play an important role in the sarcomere of striated muscle.

In the present study, we provide the first evidence that KASH-less nesprin-2 isoforms are novel sarcomeric scaffold proteins in cardiac muscle. We confirmed the interactions between nesprin-2/telethonin (titin-cap or T-cap) and nesprin-2/four and half LIM domain (FHL)-2 and showed that the nesprin-2/telethonin interaction is regulated by telethonin phosphorylation status. Importantly we also showed that binding between nesprin-2/telethonin and nesprin-2/FHL-2 was impaired by nesprin-2, telethonin, and FHL-2 mutations identified in patients with EDMD with DCM and patients with hypertrophic cardiomyopathy (HCM), implicating these complexes as key to cardiac function.

## Results

### Nesprin-2 isoforms are upregulated in embryonic mouse heart development

**Figure 1:**
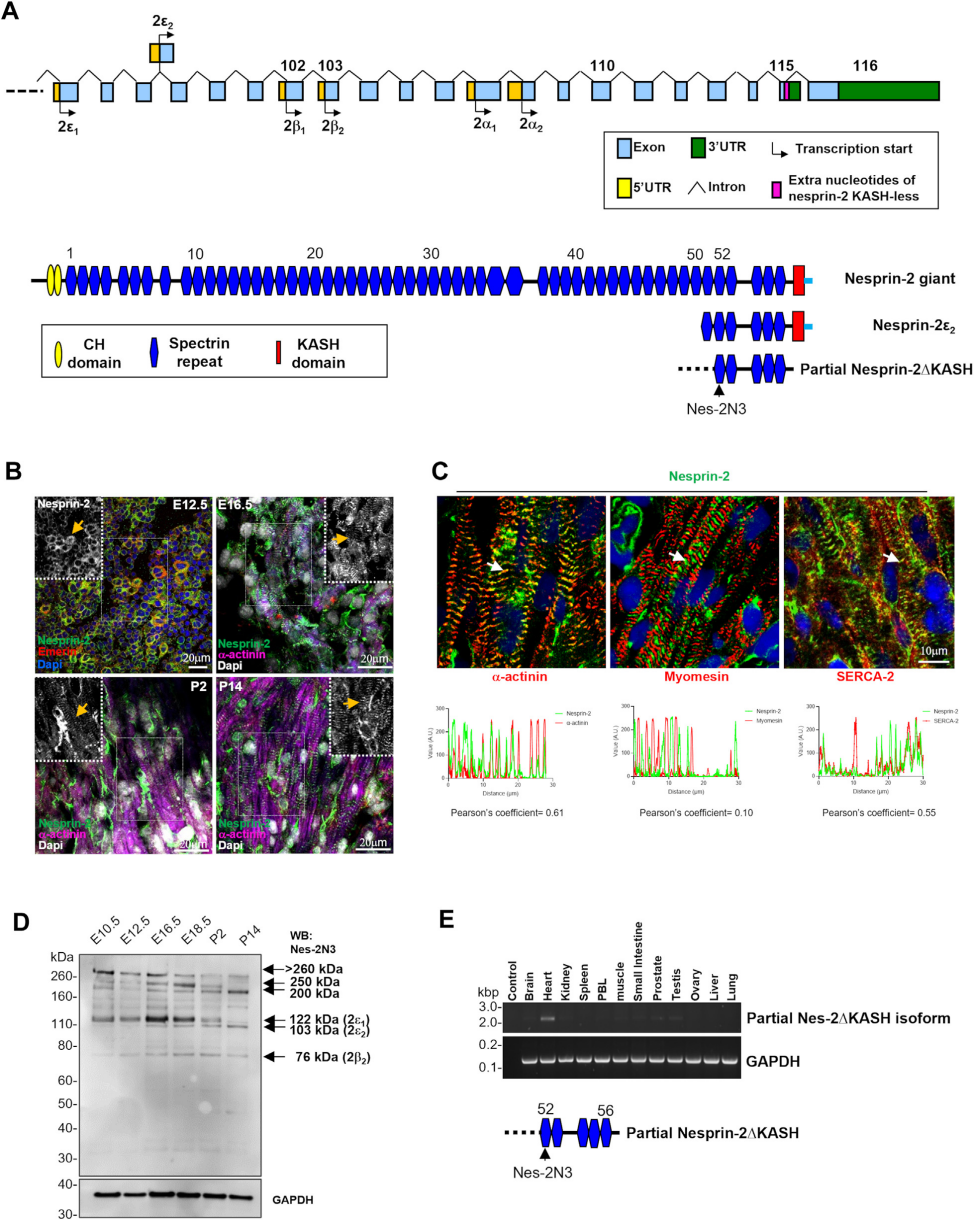
**Nesprin-2 redistributes from the NE to the sarcomere during myogenesis.***A*, schematic showing nesprin-2 giant gene and protein structure. The alternative novel stop codon is shown upstream of the KASH domain of nesprin-2 giant, which was generated by the retention of part of the intronic sequence (123 bp) immediately downstream of the penultimate exon 115, colored in *pink*). The putative partial nesprin-2 KASH-less isoform was shown with the position of nesprin-2 N3 (Nes-2N3) antibody indicated, which was specifically generated against the SR52 close to the C-terminus of nesprin-2. *B*, immunofluorescence showed nesprin-2 localised to the NE at E12.5 and was present in the sarcomere in E16.5 and during development as observed (inserts, *yellow arrowed*), colocalised with alpha-actinin (*red*) for the sarcomere and emerin (*red*) for the NE. *C*, Nesprin-2 (*green*) is present at the Z-disc and I band in the sarcomere in adult mouse cardiomyocytes, colocalised with α-actinin (*red*) and SERCA-2 (*red*). Myomesin (*red*) as a M-band marker, nuclei shown in *blue* with Dapi, Colocalisation quantification data is shown with Pearson’s coefficient for α-actinin (0.61), myomesin (0.10) and SERCA-2 (0.55), respectively. *D*, Western blot demonstrated dramatic changes in the production of nesprin-2 isoforms during mouse heart embryonic development, previously published nesprin isoforms are indicated (13, 15). *E*, qPCR verified this partial nesprin-2 KASH-less isoform that contains the SR52-53, which is exclusively present in heart tissue.

We set out to examine the nesprin-2 isoform profile during mouse muscle development using an antibody (named Nes-2N3) generated against SR52 in the nesprin-2 giant C-terminus as previously described (13) (Fig. 1*A*). Previous immunofluorescence staining using this antibody showed a sarcomeric localization for nesprin-2 in striated muscle (13). In this study, immunofluorescence staining revealed that nesprin-2 localized to the NE and nuclear periphery and co-localized with emerin at embryonic day E12.5. However, at E16.5, staining appeared in the sarcomere, co-localized with α-actinin, and was predominantly present in the sarcomere during postnatal heart development (Fig. 1*B*). In the adult mouse heart, nesprin-2 localized to the Z disc and I band, where it co-localized with α-actinin and to the sarcoplasmic reticulum co-localizing with sarcoplasmic reticulum Ca^2+^-ATPase (SERCA-2) (Fig. 1*C*). Western blot analysis using protein lysates from mouse heart tissue showed that at day E10.5, this antibody recognised six major bands at >260, 250, 200, 122 (2ε_1_), 103(2ε_2_), and 76 (2β_2_) kDa, some of which correspond to previously described isoforms indicated in brackets. Previous work has shown that isoform 2ε_1_ is enriched in embryonic cells, 2ε_2_ is mainly in cardiac muscle while 2β_2_ is predominant in striated muscle (13, 15). During mouse embryonic development, protein levels of some bands >260, 250, and 122 (2ε_1_) kDa reduced while others 200, 103(2ε_2_) and 76 (2β_2_) kDa gradually increased (Fig. 1*D*). *In vitro* myoblast differentiation confirmed changes in band sizes during maturation (Fig. S1*A*). Cell fractionation showed that the 200, 103 (2ε_2_) and 76(2β_2_) kDa bands were mainly cytoplasmic while the 122 (2ε_1_) kDa band was nuclear and up-regulated during myoblast differentiation (Fig. S1*A*). Immunofluorescence also showed that nesprin-2 redistributed from the NE and nucleus in myoblasts and neonatal cardiomyocytes to the sarcomere in myotubes and adult cardiomyocytes (Fig. S1, *B* and *C*). These data are consistent with previous studies showing a developmental transition of nesprin isoforms (5) and are highly suggestive of sarcomeric isoforms of nesprin-2.

To identify potential KASH-less isoforms containing evolutionarily conserved nesprin-2 SR52, the NCBI expressed sequence tag database (EST) was blasted with consecutive, 500 bp-overlapping 1 kb nesprin-2 sequences covering the region from SR52 to the last amino acid of the nesprin-2 giant isoform cDNA containing the KASH domain (13, 16). This search identified an alternative novel stop codon upstream of the KASH domain of the nesprin-2 giant, resulting from the retention of part of the intronic sequence (123 bp) immediately downstream of the penultimate exon 115 (Fig. 1*A*, colored in pink). This generates a novel C-terminal KASH-less isoform, terminating at amino acid 6839 (arginine) and missing the last 68 amino acids of the nesprin-2 giant. This is in agreement with a predicated termination site published previously (16). qPCR verified that a partial nesprin-2 KASH-less isoform containing SR52-56 is exclusively expressed in heart tissue (Fig. 1, *A* and *E*). To identify the 5-prime end of the transcript, 5′ RACE from heart cDNA libraries using multiple gene-specific nesprin primers and nested primers designed towards either exon 102 or 103 of the region encoding SR52-53 was performed but this strategy was unable to identify the start site of this putative KASH-less isoform (Fig. 1*E*).

### Telethonin and FHL-2 are sarcomeric binding partners for nesprin-2

**Figure 2:**
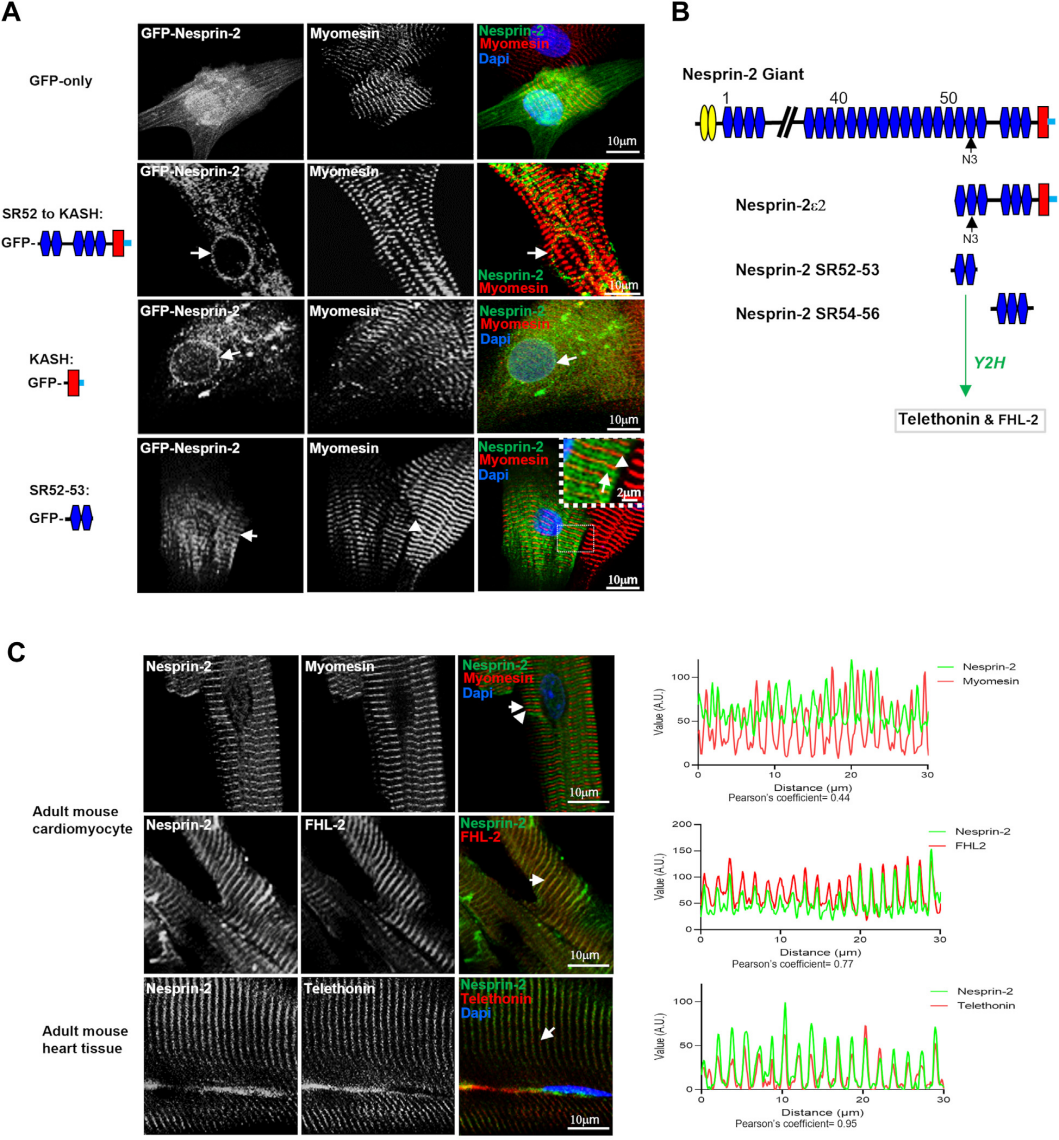
**Telethonin and FHL-2 are potential binding partners for nesprin-2 in the sarcomere.***A*, neonatal rat cardiomyocytes were transfected with either GFP empty vector or GFP-tagged nesprin-2 SR52-KASH (SR52 to last amino acid of nesprin-2 giant), KASH domain or nesprin-2 SR52-53 as shown in the schematic. 48 h after transfection, cells were fixed and stained for M-band marker myomesin (*red*). EGFP-tagged nesprin-2 SR52 to KASH and the KASH domain localized to the NE with weak staining at the sarcomere in transfected neonatal rat cardiomyocytes (two *middle panels*, *green*, *arrowed*). In contrast, EGFP-tagged nesprin-2 SR52-53 showed sarcomere staining (*bottom panel*, *green*, *arrowed*), localizing to the Z-disc and I band in contrast to myomesin-an M band protein (*arrowhead*, insert). Dapi shows the nucleus staining (*blue*). *B*, yeast two-hybrid screens on a human cardiac muscle library identified telethonin and FHL-2 are novel nesprin-2 binding partners in the sarcomere as shown in the schematic. Nesprin-2 SR52-53 (amino acids 6136–6354 of the full length of nesprin-2 giant) and SR54-56 (amino acids 6461–6781) were cloned into the GBDKT-7 matchmaker bait vector (Clontech) as bait plasmids. *C*, immunofluorescence staining showed nesprin-2 mainly localizes to the Z-disc and I band of the sarcomere in both adult mouse cardiomyocytes and heart tissue using Nes2-N3 (*green*), telethonin (red) and FHL-2 (*red*) and myomesin (*red*) antibodies. Myomesin as an M-band marker for the sarcomere. Colocalization quantification is shown with Pearson’s coefficient for telethonin (0.95), FHL-2 (0.77), and myomesin (0.44) respectively.

To observe the impact of removing the KASH domain on nesprin-2 subcellular localization and investigate the region responsible for sarcomeric targeting, we generated a series of domain-specific EGFP-tagged constructs to regions containing the C-terminal region of the nesprin-2 giant: i) SR52-KASH (the region containing SR52 to the end of the KASH domain of nesprin-2 giant); ii) SR52-53 (the targeting site for the nesprin-2 N3 antibody); and iii) the NE targeting KASH domain. When transfected into neonatal rat cardiomyocytes, both EGFP-tagged nesprin-2 SR52-KASH and KASH predominantly localized to the NE with weak sarcomeric staining (Fig. 2*A*), consistent with KASH containing isoforms as previously described (1). In contrast, EGFP-tagged nesprin-2 SR52-53, lacking SR54-56 and the KASH domain, showed mainly sarcomeric staining (Fig. 2*A*), localizing to the Z-disc and I band but not the M band protein as indicated by myomesin staining. This suggests that nesprin-2 isoforms lacking the KASH domain may target the sarcomere by C-terminal SR52-53.

To elucidate the functional role of nesprin in the sarcomere, a yeast two-hybrid screen of a human cardiac muscle cDNA library was performed using the nesprin-2 SR52-53 (amino acids 6136–6354 of the full length of nesprin-2 giant) and SR54-56 (amino acids 6461–6781) as baits. A number of potential sarcomeric binding partners, known to reside near or at the Z-disk complex, were initially confirmed after colony re-screening. Two of these potential binding partners were Tcap/telethonin and FHL-2 (Figs. 2*B* and S2). Four full-length clones of telethonin and two clones of FHL-2 encoding amino acids 85 to 279 were identified as interacting with nesprin-2 SR52-53. Immunofluorescence staining showed that nesprin-2 co-localized with telethonin and FHL-2 at the Z-disc and I band of the sarcomere in adult mouse cardiomyocytes and heart tissue (Fig. 2*C*).

### Telethonin is a novel sarcomeric binding partner for nesprin-2

**Figure 3:**
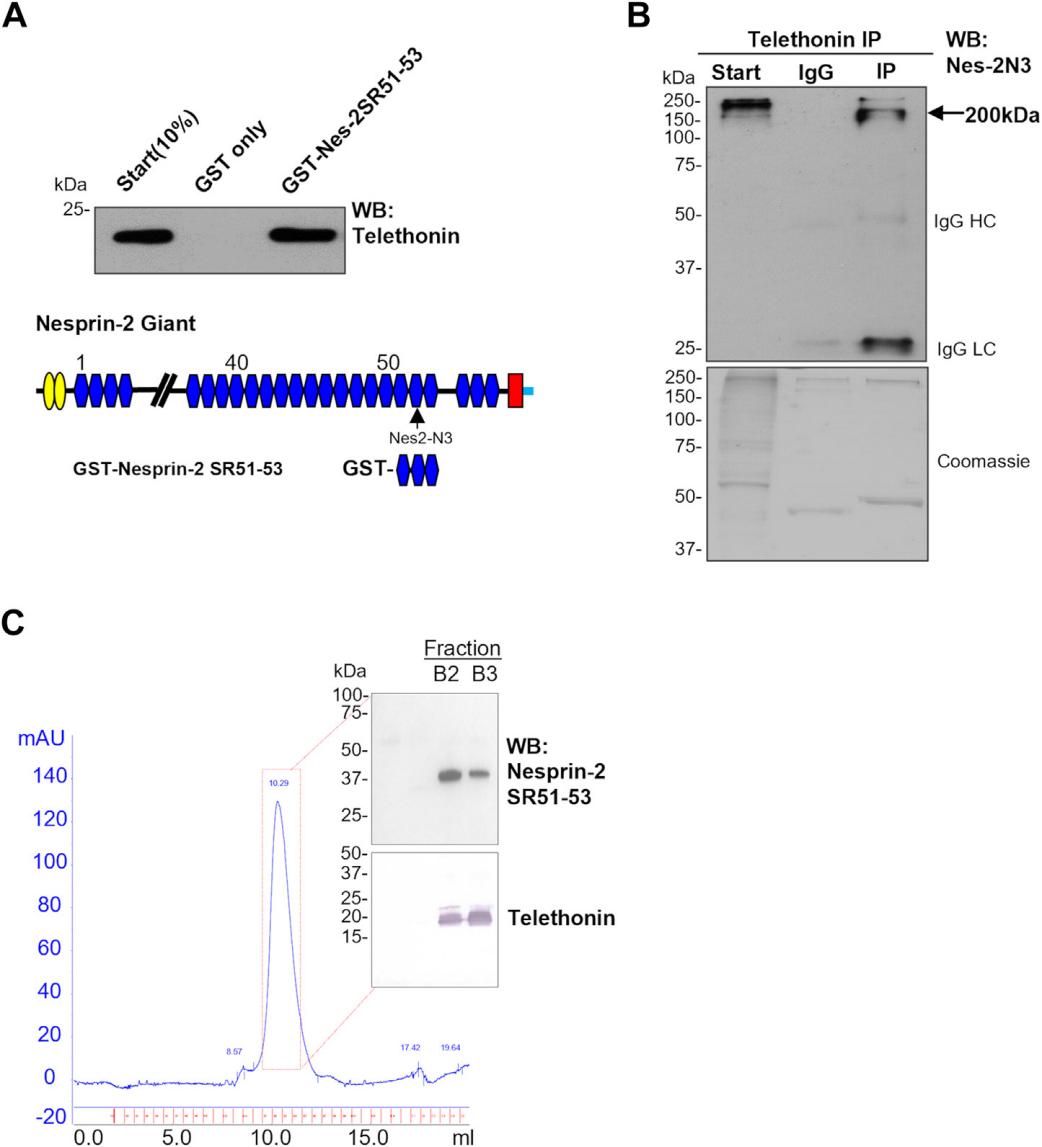
**Confirmation of the binding between nesprin-2 and telethonin.***A*, GST pull-down assay showed the interaction between nesprin and telethonin by using GST-nesprin-2 SR51-53 beads and purified His-tagged telethonin recombinant protein. *B*, IP showed telethonin antibody pull-down nesprin-2 (∼200 kDa) by using nesprin-2 N3 (Nes-2 N3) antibody and protein lysate from neonatal rat heart tissue, *C*, Co-elution of bacterially expressed and purified His-telethonin and nesprin-2 SR51-53 (no tag) by size exclusion chromatography. The fractions 101 to 112 from Superdex 200 16/60 (Fig. S3) were pooled and loaded to a S75 10/300 column. Only one peak was eluted at 10.29 ml. Western blot confirmed this single peak elution is corresponding to a 60 kDa protein (containing both nesprin-2 SR51-53: 40.51 kDa, and telethonin:19 kDa), shown by the inserts (*top*: nesprin-2 N3 antibody and *bottom*: telethonin antibody. B2 and B3 correspond to the fractions of the single peak.

We next verified the interaction between nesprin and telethonin using an extended GST nesprin-2 C-terminal SR51-53 (amino acids 6018–6354) construct. The addition of SR51 was required to maintain the stability of the bacterially expressed GST-fusion protein. Western blot analysis confirmed that the GST-nesprin-2 SR51-53 protein could precipitate His-tagged recombinant telethonin protein (Fig. 3*A*). Next, we performed immunoprecipitation experiments using neonatal heart tissue and confirmed that a telethonin antibody could precipitate a major 200 kDa nesprin-2 band (Fig. 3*B*), corresponding to the putative sacromeric isoform (Fig. 1*E*).

We next sought to confirm the interaction by co-expressing of telethonin and nesprin-2. Telethonin was cloned into a pET-Duet plasmid with an N-terminal 6x Histidine tag and nesprin-2 SR51-53 was cloned into pCDF without a tag. Both nesprin-2 SR51-53 and His-telethonin were co-expressed in BL21(DE3)pLysS *E.coli* strain, purified with a Nickel affinity column, and co-eluted by size exclusion chromatography (SEC). Western blot confirmed a single peak eluting at 10.29 ml, corresponding to a 60 kDa protein complex (nesprin-2 SR51-53: 40.51 kDa, telethonin:19 kDa, which corresponds to a 1:1 molecule ratio) (Figs. 3*C* and S3). In addition, His-telethonin and His-nesprin-2 SR51-53 were expressed and purified separately, then Native Page Novex gel analysis showed a band shift migration when telethonin was incubated with nesprin-2 SR51-53 together, indicating that telethonin aggregation is reduced in the presence of increasing concentrations of nesprin-2 SR51-53 (Fig. S4). These data suggest that nesprin-2 SR51-53 increased telethonin solubility, confirming that telethonin is a novel interacting partner of the SR51-53 regions of nesprin-2.

### Phosphorylation of telethonin regulates nesprin interaction

**Figure 4:**
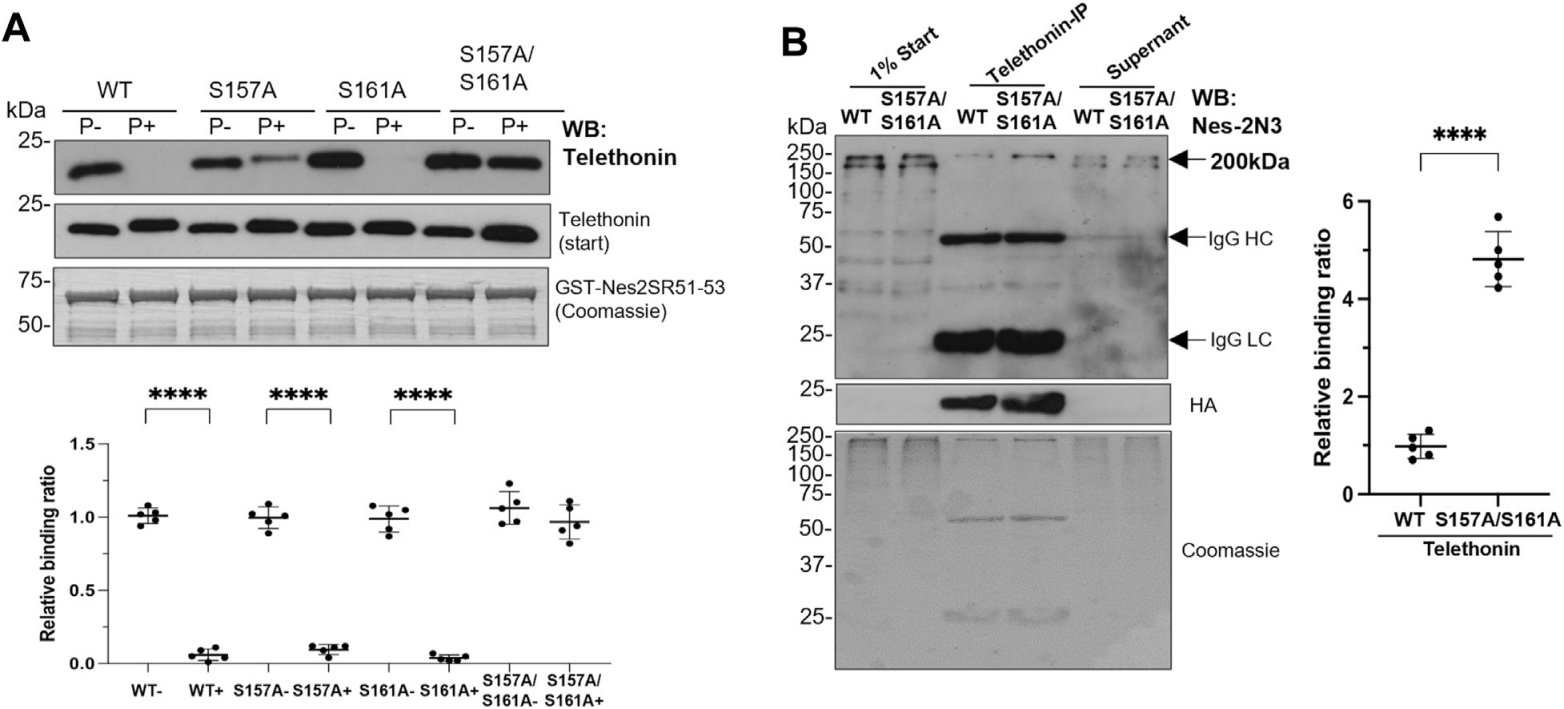
**The binding between nesprin-2/telethonin is tightly regulated by telethonin phosphorylation.***A*, HA-tagged WT and S157/161A mutated telethonin protein with or without PKD pre-treatment were incubated with GST-nesprin-2 SR51-53 beads for a GST pull-down assay. The nesprin binding for wild-type (WT) or mutant recombinant telethonin proteins was quantified by densitometry with respect to the input material and expressed as a ratio of the value obtained for unphosphorylated WT telethonin. Five independent experiments were performed shown as mean ± SD, ∗∗∗∗*p* < 0.0001. F (7, 32) =209.5 using one-way ANOVA analysis; Bonferroni post-hoc tests showed ∗∗∗∗*p* < 0.0001 for WT-*versus* WT+, S157- *versus* S157+, S161- *versus* S161+ respectively. *B*, NRCs were infected with adenovirus carrying HA-tagged WT telethonin or S157/161A double mutants. Protein lysates were harvested 48 h after adenoviral infection and then subjected to immunoprecipitation using a telethonin antibody. Western blot showed nesprin-2 was significantly pulled down in neonatal cardiomyocytes with over-expressed unphosphorylatable S157/161A telethonin. HA antibody used on the same blot showed an equal amount of HA-tagged telethonin pulled down by telethonin antibody. The intensity of about ∼200 kDa nesprin pull-down bands was measured by densitometry with respect to the input material and relative binding to nesprin expressed as a ratio of the value obtained between mutant and WT telethonin. IP was repeated for five times. Student’s *t* test (unpaired, two-tailed) showed a significantly higher binding strength between nesprin and S157/161A double mutated telethonin than the WT. ∗∗∗∗*p* < 0.0001.

Telethonin is phosphorylated by titin kinase at Ser157 and protein kinase D (PKD) at both Ser157 and Ser161. Induction of an alanine mutation at either or both sites can lead to partially or totally unphosphorylatable telethonin variants (17). Therefore, we investigated whether telethonin phosphorylation could affect nesprin binding. HA-tagged WT and mutant telethonin (S157A and/or S161A) with or without PKD pre-treatment were incubated with GST-nesprin-2 SR51-53 beads. GST pull-down revealed that the S157A and S161A mutations do not affect the interaction between telethonin and nesprin-2. However, the phosphorylation of WT telethonin dramatically reduced the binding to nesprin-2 SR51-53 (Fig. 4*A*). Furthermore, there was very limited binding between nesprin-2 and either S157A or S161A mutants pre-treated with PKD, indicating phosphorylation of either Ser-161 or Ser-157 is sufficient to inhibit nesprin-2/telethonin interactions (Fig. 4*A*). The binding between nesprin and the unphosphorylatable S157A/S161A double mutants was not affected by PKD pre-treatment (Fig. 4*A*), further suggesting that the interaction between nesprin-2 and telethonin is regulated by the phosphorylation status of telethonin.

To further confirm that the phosphorylation status of telethonin plays a role in regulating nesprin binding, neonatal rat cardiomyocytes were infected with adenovirus carrying HA-tagged WT telethonin or the S157A/S161A double mutants. Immunoprecipitation showed that nesprin-2 was more efficiently precipitated by a telethonin antibody from neonatal rat cardiomyocytes expressing the unphosphorylatable S157A/S161A telethonin double mutant than WT control (Fig. 4*B*) further suggesting that the phosphorylation of telethonin reduces nesprin-2 binding.

### FHL-2 is also a novel sarcomeric binding partner for nesprin-2

**Figure 5:**
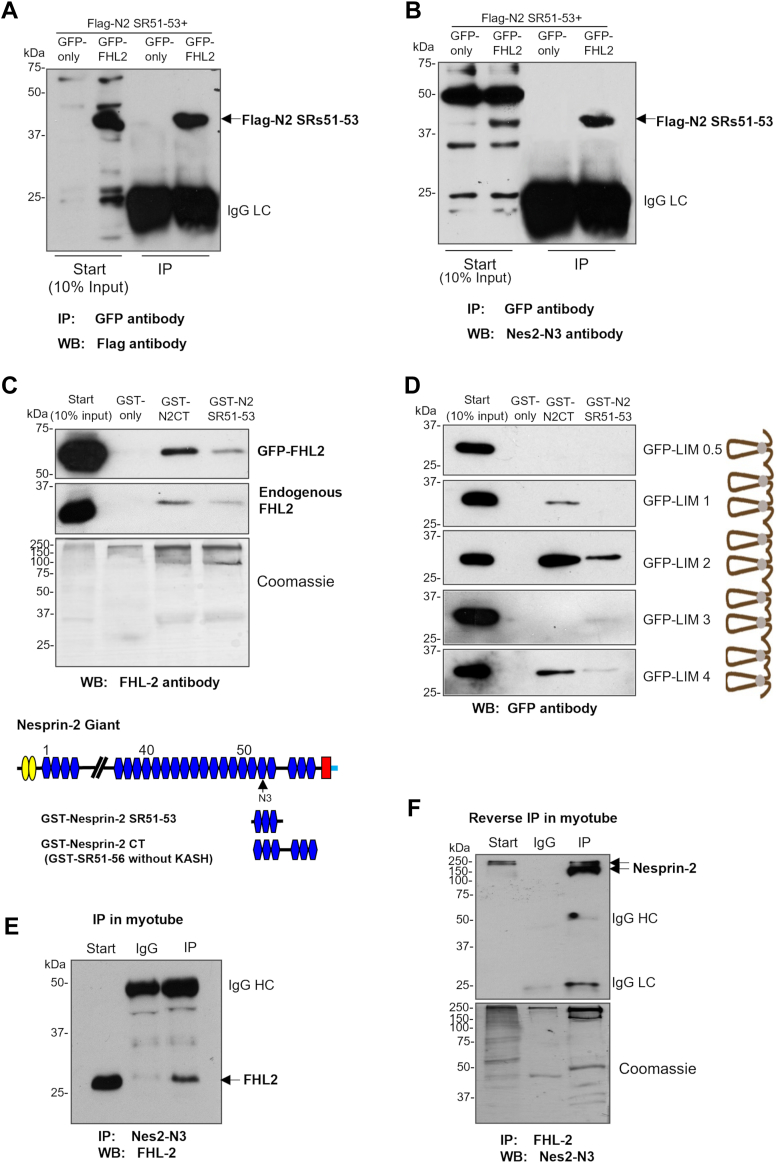
**FHL-2 is also a novel sarcomeric binding partner for nesprin-2.***A* and *B*, overexpression of Flag-tagged nesprin-2 and GFP-FHL-2 proteins in C2C12 myoblasts and then immunoprecipitation with GFP antibody. Western blotted with both Flag antibody and Nes-2N3 antibody respectively, showed nesprin-2 and FHL-2 binding. *C*, GST pull-down GFP-FHL-2 using GST-nesprin-2 SR51-53 and GST nesprin-2 CT (SR 51-56 without KASH). *D,* Functional binding domains of nesprin-2 and FHL-2 were mapped using nesprin-2 and FHL-2 deletion constructs shown by the schematic. *E* and *F*, immunoprecipitation in C2C12 myotube lysates with both nesprin-2 N3 and FHL-2 antibodies confirmed that FHL-2 is a novel nesprin-2 binding partner.

Nesprin-2 also co-localized with FHL2 in the I band and Z disc. To confirm that FHL-2 is a novel nesprin-2 binding partner, we co-expressed the FLAG-tagged nesprin-2 SR51-53 and GFP-tagged FHL-2 in C2C12 myoblasts. These cells were chosen because of easy accessibility and higher transfection efficiencies compared to neonatal rat cardiomyocytes and both nesprin-2 and FHL-2 are also expressed in striated skeletal muscle cells. Immunoprecipitation confirmed that FLAG-tagged nesprin-2 SR51-53 was efficiently precipitated by GFP-FHL-2 (Fig. 5, *A* and *B*). To map the functional binding domains of nesprin-2 and FHL-2, we used deletion constructs and GST-pulldown assays. This approach revealed that SR51-53 of nesprin-2 predominantly binds to the LIM-2 domain of FHL2 (Fig. 5, *C* and *D*). Using C2C12 myotube lysates, immunoprecipitation confirmed the nesprin-2 and FHL-2 interaction and reverse immunoprecipitation demonstrated that an FHL-2 antibody could precipitate a major 200 kDa nesprin-2 band (Fig. 5, *E* and *F*), equivalent to the putative sarcomeric isoform shown in Figure 1*E*, highly indicative of a nesprin-2/FHL-2 interaction.

### Nesprin-2 binding is impaired in patients with EDMD with DCM or HCM harboring nesprin-2, telethonin, or FHL-2 mutations

**Figure 6:**
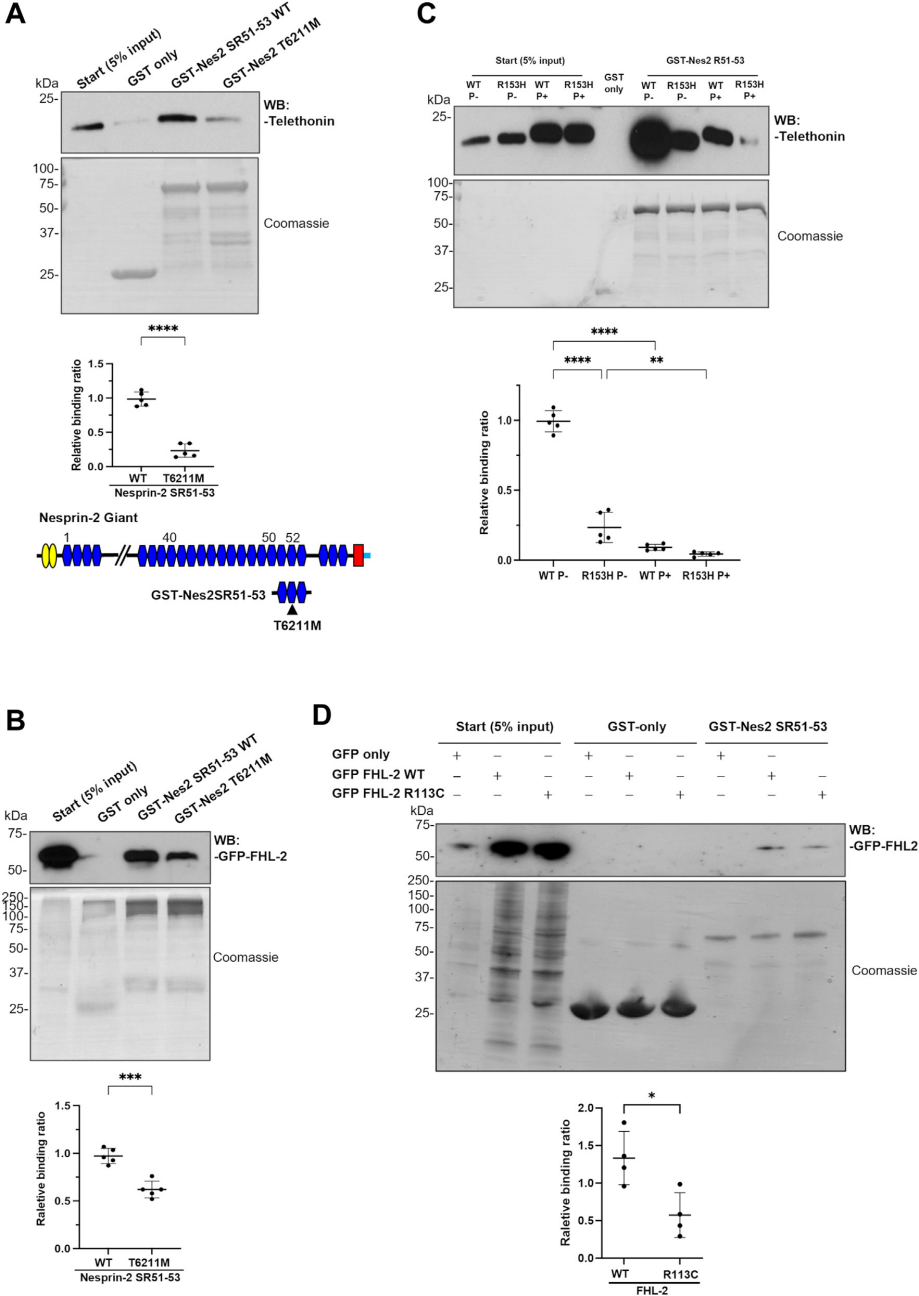
**Nesprin2/telethonin or nesprin-2/FHL-2 interaction was impaired by both nesprin-2 and telethonin or FHL-2 mutants.** GST-nesprin-2 SR51-53 WT and mutant T6211M proteins were incubated with His-tagged telethonin recombinant protein (*A*) or GFP-tagged FHL-2 protein (*B*) for GST pull-down assay. Coomassie blue staining of the gel showed an equal amount of GST-nesprin-2 SR51-53 WT and T6211M beads were used. The His-telethonin or GFP-FHL-2 binding for GST-Nesprin-2 SR51-53WT and GST-nesprin-2 SR51-53 T6211M was quantified by densitometry and expressed as a ratio of the value to show the relative binding. The GST pull-down was repeated five times, and Student’s *t* test (unpaired, two-tailed) confirmed the T6211M mutation significantly reduced nesprin binding with telethonin (∗∗∗∗*p* < 0.0001) or FHL-2 (∗∗∗*p* = 0.002). The schematic showed where T6211M is located (*A*). *C*, his-tagged WT and R153H mutated telethonin with or without PKD pre-treatment were incubated with GST-nesprin-2 SR51-53 beads for a GST pull-down assay. Coomassie blue staining of the SDS-PAGE gel showed an equal amount of GST-nesprin-2 SR51-53 beads were used for each pull-down. The nesprin interaction with different telethonin protein samples was measured by densitometry with respect to the input material and relative binding to nesprin expressed as the ratio of the nesprin binding to the unphosphorylated WT telethonin. Five independent experiments were performed shown as mean ± SD, ∗∗∗∗*p* < 0.0001, F (3, 16) = 218 using one-way ANOVA analysis; Bonferroni post-hoc tests showed ∗∗∗∗*p* < 0.0001 for WT P- *versus* WT P+, WT P- *versus* R153H P-; ∗∗*p* = 0.0015 for R153H P- *versus* R153H P+ respectively. *D*, GFP-tagged FHL-2 WT, and R113C mutant were incubated with GST-nesprin-2 SR51-53 beads for a GST pull-down assay. Coomassie blue staining of the SDS-PAGE gel showed an equal amount of GST-nesprin-2 SR51-53 beads were used for each pull-down. The nesprin binding for WT or mutant FHL-2 proteins was quantified by densitometry with respect to the input material and expressed as a ratio of the value obtained for WT FHL-2. Four independent experiments were performed shown as mean ± SD, ∗*p* < 0.0179 using Student’s *t* test (unpaired, two-tailed).

To gain insights into the functional significance of these interactions, we next examined binding associations in the presence or absence of disease-causing mutations. A T6211M mutation in SR52 of nesprin-2 giant, located in the telethonin and FHL2 binding region, was identified in patients with EDMD with DCM (3). An R153H mutation, close to the two phosphorylation sites (amino acid 157 and 161) of telethonin, was identified at the C-terminal region of telethonin in hypertrophic cardiomyopathy (HCM) patient (18), and an R113C mutation in the nesprin-2 binding region was identified in the LIM-2 domain of FHL-2 in an EDMD patient with conduction defect (Fig. S5). GST pull-down assays showed the interaction between nesprin-2/telethonin (Fig. 6*A*) and nesprin-2/FHL2 (Fig. 6*B*) was significantly reduced in the presence of the nesprin-2 T6211M mutation. Both the telethonin R153H mutant (Fig. 6*C*) and the FHL-2 R113C mutant (Fig. 6*D*) also significantly reduced the binding between nesprin-2/telethonin and nesprin-2/FHL2. Telethonin phosphorylation status further impaired its binding to nesprin-2 (Fig. 6*C*), suggesting misregulation of these interactions is associated with muscle cell dysfunction.

### Nesprin-2 localization is altered in cardiomyocyte hypertrophy

**Figure 7:**
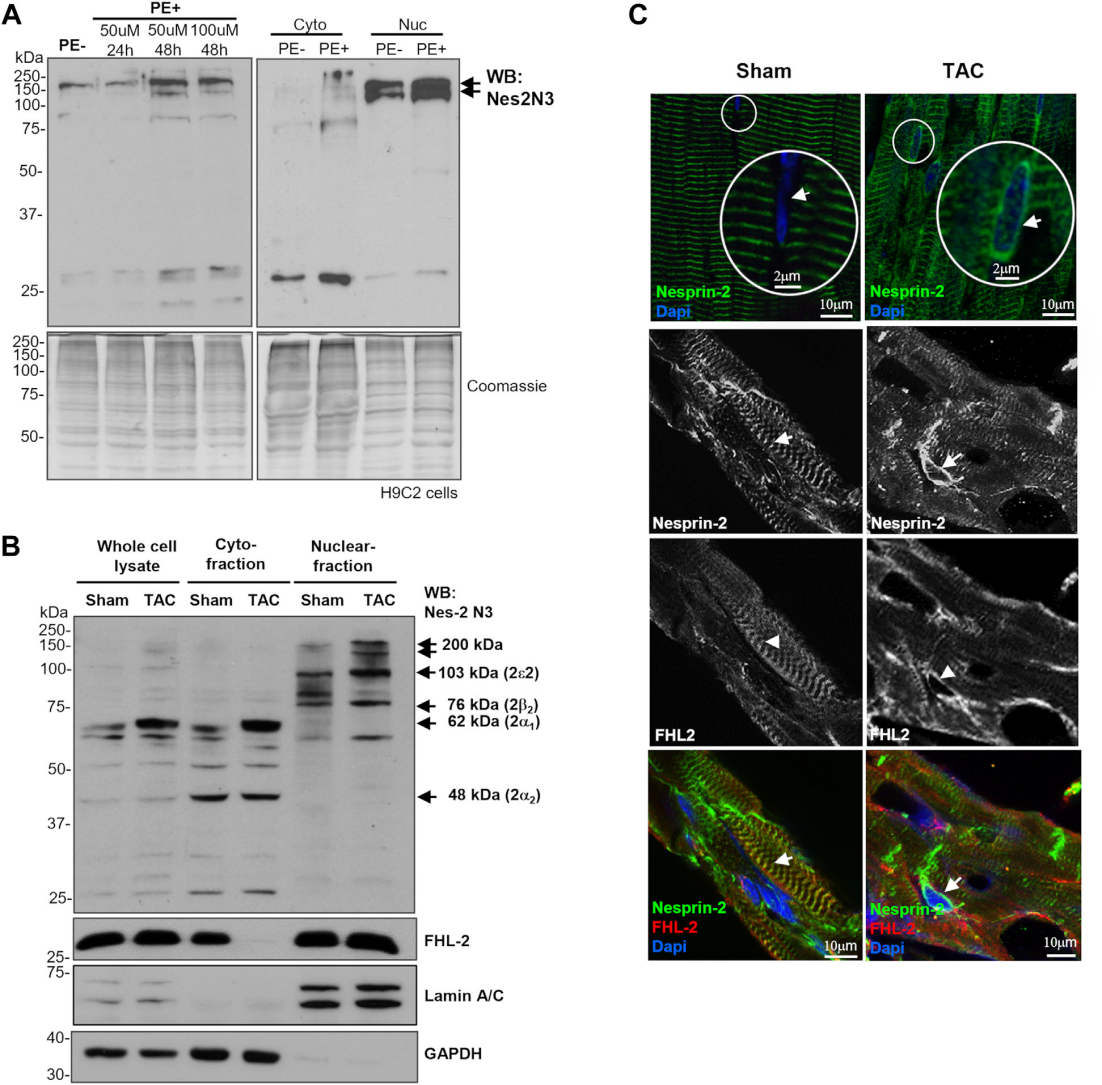
**A potential role of nesprin-2 in regulating hypertrophic response.***A*, Western blot showed a ∼200 kDa nesprin-2 isoform was significantly up-regulated in the nuclear fraction of the H9C2 cells treated with phenylephrine, 25 kDa nesprin in the cytoplasmic fraction could be potential isoforms due to extensively alternative initiation and splicing or degradation products, further verification needed. *B* and *C*, Western blot and immunofluorescence showed nesprin-2 isoforms, especially those containing SR 52-53 redistributes from the sarcomere to the NE in hypertrophic cardiomyocytes in a transverse aortic constriction (TAC) mouse model (*arrowed*), and FHL-2 subcellular localization was affected shown in (*C*, *arrowhead*).

FHL2 is highly expressed in the heart throughout embryonic development and in adults. FHL2 is known to inhibit cardiac hypertrophic pathways (19). Interestingly, we observed that nesprin-2 was up-regulated and redistributed from the cytoplasm to the nucleus in embryonic rat cardiomyocyte H9C2 cells stressed with phenylephrine (Fig. 7*A*). These observations led us to test whether induction of hypertrophy *in vivo* could affect nesprin-2 homeostasis. To test this *in vivo*, we used a transverse aortic constriction (TAC) mouse model. Analysis of cardiac cell lysates by Western blot showed that bands at 200, 103(2ε_2_), and 76 (2β_2_) kDa were increased in the nuclear fraction while a 62 kDa (2α_1_) band was reduced and a 48 kDa (2α_2_) band unchanged in the cytoplasmic fraction (Fig. 7*B*). Immunofluorescence showed both nesprin-2 and FHL-2 redistributed from the sarcomere to the NE in hypertrophic cardiomyocytes (Fig. 7*C*). These data suggest that nesprin-2 may have a role in the regulation of cardiac hypertrophy.

## Discussion

In this study, we have identified nesprin-2 as a component of the Z disc and I band of the sarcomere, and a novel binding partner for both telethonin and FHL2 in cardiomyocytes. The interactions between nesprin-2/telethonin and nesprin-2/FHL2 were impaired by nesprin-2, telethonin, and FHL-2 mutations identified in EDMD with DCM and HCM patients respectively. Furthermore, the binding between nesprin-2 and telethonin was tightly regulated by the phosphorylation status of the telethonin C-terminus. These findings suggest that these interactions at the Z-disc are important for sarcomere structural organization and function.

### Change in the expression pattern of nesprin-2 during muscle development

Changes in expression levels and subcellular localisations of nesprin-1 and -2 during muscle development were first observed in an *in vitro* C2C12 myoblast differentiation system (13). A further study demonstrated that giant nesprin-1 and -2, which are predominant in fibroblasts, have low expression in both myotubes and mature muscle cells. Instead, increased expression of smaller nesprin isoforms (nesprin-1α_2_, nesprin-2α_1_, and nesprin-2ε_2_) was observed in mature skeletal and cardiac muscle cells (15). The reason for the distinctive expression patterns of nesprin-1 and -2 in muscle cells remains unclear but is likely related to tissue-specific functions of certain nesprin −1 and −2 isoforms. In this study, we identified a novel partial nesprin-2 isoform lacking the KASH domain and containing SR52-53 of the nesprin-2 giant. Importantly, this KASH-less isoform can target to the sarcomere in cardiomyocytes. Our data showed that a major 200 kDa nesprin-2 isoform containing SR52-53 is dramatically up-regulated during cardiac muscle development and was able to bind to the sarcomeric proteins telethonin and FHL-2. This suggests for the first time that nesprin-2 isoforms may also perform scaffolding roles in the striated muscle sarcomere, particularly in cardiac muscle. These sarcomeric scaffolding functions of nesprin-2 are distinct from the well-established roles of nesprin-2 KASH containing isoforms present at the NE.

### Nesprin-2 may participate in maintenance of the sarcomere organisation and function

Our data showed nesprin-2 as a binding partner for telethonin in cardiomyocytes. Telethonin is a 19 kDa protein encoded by the *TCAP* gene, which is expressed almost exclusively in cardiac and skeletal muscle (17). Mutations in *TCAP* are causally associated with both skeletal and cardiac myopathies (20). The N-terminal region of telethonin forms a unique β-sheet structure binding to two titin molecules, thus anchoring titin in the sarcomeric Z-disc and provides the assembling and anchoring framework for the huge titin filaments (21, 22). Telethonin is phosphorylated by titin kinase at Ser157 and PKD at both Ser157 and Ser161, suggesting it plays a key role in sarcomere assembly and mechanotransduction (17). Telethonin also anchors other Z disc proteins such as muscle LIM-protein to form the Z-disc mechanical stretch sensor machinery, which allows rapid adaption to elevated mechanical stress in cardiomyocytes (23). Defects in the mechanosensory machinery could give rise to cardiomyopathy (18, 23, 24). In this study, we identified nesprin-2 as a novel binding partner for telethonin, with binding tightly regulated by phosphorylation at the C-terminus of telethonin. Endogenous telethonin is in a constitutively bis-phosphorylated form, with only small proportions of the protein present in a mono- and non-phosphorylated state in wild-type rat and mouse myocardium (17). Expression levels of the under-phosphorylated form of telethonin are increased during pressure overload-induced remodeling of mouse hearts (25). Interestingly, we observed more nesprin-2 in the cardiomyocyte sarcomere and nuclear envelope/nucleus after stress (PE treatment and TAC). With increasing diastolic wall stress, the elastic region of titin is stretched, affecting the conformation of the titin/telethonin complex at the Z-disc complex (21, 22). This can, in turn, change the phosphorylation status of telethonin, which would then regulate its association with nesprin-2 and possibly other binding partners.

We demonstrated that both the T6211M nesprin-2 mutation identified in patients with EDMD and DCM and the R153H telethonin mutation identified in an HCM patient significantly altered the interaction between nesprin-2 and telethonin. Mutations in nesprin-2 and telethonin, by affecting their binding either directly or *via* phosphorylation, could impair this sarcomeric function under strain particularly as telethonin is a part of the Z-disc mechanical stretch sensor machinery (22, 23, 26). More experiments are now required to understand how disrupting these interactions links to pathology.

### Nesprin-2 may participate in regulating hypertrophic response in cardiomyocytes

We identified nesprin-2 as a binding partner for FHL-2 in cardiomyocytes. FHL-2 is a 32 kDa protein, that can inhibit the cardiac hypertrophic response to beta-adrenergic stimulation (27). LIM domain proteins have been shown to shuttle to the nucleus and are attractive candidates to provide a connection between stretch sensing at the level of the sarcomere and signaling modules leading to transcriptional changes and cardiomyocyte growth (28). Our data showed nesprin-2 binds to FHL2, and that this interaction was disrupted by both the T6211M nesprin-2 mutation and R113C FHL2 mutation. Furthermore, nesprin-2 was redistributed from the sarcomere to the nucleus under stress and nesprin-2 isoforms, especially those containing SR52-53 (about 200 kDa isoform, 2ε_2_ and 2β_2_) were significantly up-regulated in hypertrophic cardiomyocytes, suggesting nesprin proteins may reiterate their developmental expression under stress. It would be interesting to investigate if the interaction between nesprin-2 and FHL2 plays a role in cardiac mechanical sensing and hypertrophy and thus contributes to cardiomyopathies.

Taken together our data suggests there is an alternative pathway for nesprin-2 mutations in causing muscle cell dysfunction, leading to EDMD, DCM, and HCM, in addition to LINC disruption at the NE. The functions and regulation of nesprin-2 in the cardiac sarcomere warrant further investigation. Further studies are also required to elucidate the precise mechanisms mediating the biochemical interactions between nesprin-2, FHL-2, and telethonin (with/without phosphorylation) in the sarcomere. However, more work is now needed to understand the complexities of their interactions and whether FHL2 and telethonin can interact with nesprin-2 at the same time or competitively. Understanding the spatial/temporal dynamics of their interactions and how they might change in response to loading will provide valuable mechanistic insights into cardio/muscular myopathies.

## Experimental procedures

### Animal studies (mouse embryonic development)

All procedures were performed in accordance with the Guidance on the Operation of the Animals (Scientific Procedures) Act 1986 (UK Home Office) under animal license PP3948135 approved by the UK Home Office. For the isolation of adult mouse hearts, mice were sacrificed by cervical dislocation and exsanguination.

### Isolation, culture, and transfection of neonatal rat cardiomyocytes

Neonatal rat cardiomyocyte (NRC) isolation was performed using a Worthington Neonatal Cardiomyocyte Isolation System (Worthington Biochemical Corp) following the manufacturer's instructions. NRCs were isolated from 1- or 2-day-old neonatal Sprague–Dawley rats. Hearts were then washed, excised, minced and enzymatically digested at 37 °C with ADS buffer [116 mmol/L NaCl, 20 mmol/L HEPES, 0.8 mmol/L NaH2PO4, 5.6 mmol/L glucose, 5.4 mmol/L KCl, 0.8 mmol/L MgSO4] containing collagenase (57.5 U/ml) and pancreatin (1.5 mg/ml). The suspension was pre-plated to remove contaminating cells, before being cultured on gelatin (Sigma) pre-coated 35 mm petri dishes with a density of 2 × 10^5^ cells/ml. Cells were allowed to adhere for 24 h, and then transfected using Escort III (Sigma) following the manufacturer’s instruction as described previously (29).

### Isolation and culture of adult rat cardiomyocytes

Ventricular myocytes were isolated from the hearts of adult male Wistar rats (200–250 g, B & K Universal Ltd) (17). Briefly, hearts were excised from terminally anaesthetised and heparinised (60 mg/kg sodium pentobarbitone and 100 U sodium heparin i.p.), hearts were initially perfused for 5 min with modified HEPES-Krebs solution (pH 7.3 at 37 °C) containing NaCl (130 mmol/L), MgCl2 (4.5 mmol/L) NaH2PO4 (0.4 mmol/L), CaCl2 (0.75 mmol/L), HEPES (4.2 mmol/L), taurine (20 mmol/L), creatine (10 mmol/L) and glucose (10 mmol/L). Hearts were then perfused in Langendorff mode with Ca^2+^-free HEPES-Krebs solution containing EGTA (1 mmol/L) for 4 min, and subsequently with HEPES-Krebs solution containing CaCl2 (0.1 mmol/L) and 125 U/ml Type II collagenase (Worthington Biochemical Corporation) for 8 min. All solutions were maintained at 37 °C and gassed with 100% O_2_. Following perfusion, the heart was removed from the cannula and the non-ventricular and atrial tissue carefully removed, before the ventricles were cut into small pieces and incubated in 30 ml of the collagenase solution gassed with 100% O_2_ for a further 10 min at 37 °C, with regular trituration. Isolated myocytes were separated from undigested tissue by filtering through nylon gauze (200 μM pore size) and allowed to settle into a loose pellet for 10 min before being washed with HEPES-Krebs solution containing CaCl_2_ (0.5 mmol/L) and 1% BSA. A further settling and wash step was performed during which the CaCl_2_ concentration was increased to 1 mmol/L. Isolated myocytes were washed at room temperature with modified M199 culture medium containing creatine (2 mmol/L), carnitine (2 mmol/L), and taurine (5 mmol/L), plus 100 IU/ml penicillin/streptomycin, pelleted by brief centrifugation and again resuspended in fresh modified M199 medium. ARVM were then pipetted into pre-laminated wells of plastic 6-well culture plate and allowed to adhere for 90 min in a humidified incubator (37 °C, 5% CO_2_). The culture medium was replaced with fresh modified M199 medium prior to adenoviral infection.

### PCR of human nesprin-2 KASH-less and 5′ rapid amplification of cDNA ends

Tissue distribution for partial nesprin-2 KASH-less isoforms was generated using a multi-tissue cDNA panel and PCR primers designed with 3′- primer targeted to the intronic region downstream of exon 115 (5′- CAGCCCTTTCCAGACAAAAG-3′) and the 5′- primer within a constitutively present exon 102 that encodes SR52-53 (5′-TGGCAGAAGTTTTTAGACGACTA-3′) (13, 16). To try to generate 5′ end of nesprin-2 KASH-less isoforms, 5′ rapid amplification of cDNA ends (RACE) was performed from heart cDNA libraries using gene-specific nesprin primer (5′- CCTGCTGGCCGTGTCTGTGCGGTTCTCCC-3′) and nested primer (5′-GAGCTCCAGCTGAGTGAGCCGCTCATG-3′) designed towards either exon 102 or 103 close to the region encoding SR52-53 (amino acids 6136–6354 of the full length of nesprin-2 giant) and an Advantage-GC2 PCR Kit (Clontech) (1, 13). PCR fragments were cloned into pGEM-Easy (Promega) for sequencing verification (Source BioScience).

### Plasmid constructs and site-directed mutagenesis

Human cDNAs for enhanced GFP (EGFP)- nesprin-2 SR52-53 (amino acids 6136–6354), nesprin-2 SR52-KASH (comprised the region containing SR52 to last amino acid of nesprin-2) and KASH domain constructs were amplified using a high-fidelity GC-rich PCR kit (Roche) and inserted in-frame into Bgl II or BspE I and Sal I sites of the pEGFP-C1 vector (Clontech). Nesprin-2 SR51-53 was also cloned into a pCMV-Tag 2C vector containing an N-terminal Flag tag (Agilent Technologies) using BamH I and Xho I sites. Nesprin-2 SR51-53 (amino acids 6018–6354, including SR51 for GST-protein stability) and nesprin-2 CT (SR51-56, amino acids 6018–6781, without KASH) were amplified and inserted in-frame into EcoR I and Sal I sites of a modified GST (pGEX4T-1) construct (30) respectively for GST pull-down assay. The GST-nesprin-2 SR51-53 mutant constructs (T6211M) were generated using a QuikChange XL site-directed mutagenesis kit (Stratagene) (3). GFP-FHL-2 R113C mutant construct was generated from GFP-FHL-2 WT using this mutagenesis kit. GFP-tagged constructs for FHL-2 WT, FHL-2 LIM-0.5, 1, 2, 3, and 4, domains were previously generated (31).

### Yeast two hybrid screening

Nesprin-2 SR52-53 (amino acid 6136–6354 of the full length of nesprin-2 giant) and SR54-56 (amino acids 6461–6781) were cloned into the GBDKT-7 matchmaker bait vector (Clontech) and transformed into the yeast strain AH109 (Clontech). Bait plasmids were tested for self-activation and then used for screening in the cardiac muscle library (cloned into pACT2, Clontech) as described before (7).

### Nesprin and telethonin co-expression

Nesprin-2 SR51-53 (amino acids 6018–6354 of the full length of nesprin-2 giant) was cloned into CDF DUET-1 plasmid (Novagen) with restriction Nde1/Xho1 sites without a tag. Full-length telethonin was cloned in pET DUET-1 (Novagen) with restriction sites BamH1/Xho1 with an N-terminal His-tag. Both pDuet (His-telethonin) and pCDF (nesprin) constructs were transformed together in BL21(DE3)pLysS *E.coli* strain at a concentration of 6 ng of pET Duet plasmid and mixed with 15 ng of pCDF plasmid. Expression was done in LB medium containing chloramphenicol 32 μg/ml, ampicillin 100 μg/ml, and streptomycin 10 μg/ml. The bacterial cells were incubated at 37 °C until OD_600_ was 0.5 and then induced by 0.4 mM IPTG, and overnight incubation at 18 °C at 200 rpm.

### Nesprin-telethonin complex purification

After overnight incubation, cells were centrifuged at 4000*g* for 15 min at 4 °C. Pellets were washed once with cold PBS and centrifuged again. The pellets were stored at −80 °C. Pellets were thawed and resuspended in a lysis buffer (50 mM Tris/HCl pH7.5; 150 mM NaCl; 20 mM β-Mercaptoethanol, 1 mM PMSF, and DNase at 10 μg/ml), the mixture was passed twice through the smasher and then incubated on ice for 20 min. The homogenate was centrifuged at 20,000 rpm in a JA20 Beckman rotor for 30 min. The soluble fraction was transferred to a tube and imidazole was added to a final concentration of 10 mM, followed by passing through a 0.2 μM filter and loading onto 2 × 1 ml His-trap columns (GE Healthcare) in tandem equilibrated with lysis buffer containing 10 mM imidazole. Columns were washed with equilibration buffer and protein was eluted with 600 mM imidazole in equilibration buffer.

### Size exclusion chromatography

The protein elution above was loaded onto a Superdex 200 16/60 column equilibrated with a buffer of 50 mM Tris/HCl pH7.5; 150 mM NaCl; 20 mM βME. The column was run at 2 ml/min flow rate, and fractions of 2 ml were collected, and an aliquot was run on an SDS-PAGE. The peak (fractions 101–112) that contained nesprin-2/telethonin complex were pooled and loaded onto a Superdex 75 10/300 column for further purification. The column was equilibrated with the same buffer, and the βME was increased to 100 mM to reduce aggregation. The column was run at a 0.5 ml/min flow rate, and fractions were collected. The complex elutes as a single peak at 10.29 ml. An aliquot of this elution was run onto 8 to 16% SDS-PAGE and blotted to a PVDF membrane. The membrane was first probed with nesprin-2 N3 antibody and developed with HRP substrate (Thermo Fisher Scientific) and visualized on X-ray film. Then the membrane was stripped and then incubated with telethonin primary antibody (sc-25327, Santa Cruz) and a secondary mouse antibody conjugated with alkaline phosphatase, then visualized with Novex AP Chromogenic Substrate (BCIP/NBT) (Thermo Fisher Scientific).

### Native Page Novex gel analysis

Nesprin-2 SR51-53 (amino acids 6018–6354 of the full length of nesprin-2 giant) was cloned into a modified pET22B construct with an N-terminal His-tag and restriction EcoR I/Sal I sites (30). His-tagged full-length telethonin was previously generated (kind gifts from Prof Metin Avkiran, King’s College London) (17). Each construct was transformed into BL21(DE3)pLysS *E.coli* strain. Expression was done in LB medium containing chloramphenicol 32 μg/ml and ampicillin 100 μg/ml. The cells were incubated at 37 °C until OD600 was 0.5 and then IPTG was added to 0.4 mM final concentration, and overnight incubated at 18 °C at 200 rpm. His-tagged telethonin and nesprin-2 SR 51-53 were purified from inclusion bodies with Nickel affinity column using 1% Sarcosyl in phosphate buffer pH7.4, 300 mM NaCl + 30 mM DTT, with final concentration of 0.3% Sarcosyl separately. Both proteins were mixed at increasing molar ratios of nesprin:telethonin in a 25 μl reaction mix in PBS containing 0.3% Sarcosyl and incubated for 20 min at room temperature. After incubation, the loading buffer was added, and the mixture was briefly centrifuged then loaded on a Novex Native gel and run as instructed by the manufacturers (Thermo Fisher Scientific). After electrophoresis, the gel was transferred onto a PVDF membrane and probed with a telethonin antibody (sc25327, Santa Cruz). Protein was visualized using the alkaline phosphatase reaction.

### HA or His-tagged telethon WT and mutant generation

HA-tagged telethonin WT, S157A, S161A, S157/161A and His-tagged telethonin WT, R153H mutants were previously generated (kind gifts from Prof Metin Avkiran, King’s College London) (17). Briefly, Purified WT, S157A, S161A and S157/161A, R153H telethonin proteins were incubated with PKD *in vitro*. The protein samples with or without PKD treatment were loaded on a Phos-tag SDS-PAGE gel for electrophoresis and Western blotting (17).

### Adenovirus infection

For adenoviral infection, NRCs were plated in a fibronectin-coated 6-well culture plate at 1.2 × 10^5^ cells/ml. Before infection, the plating medium was replaced by culture medium and healthy attaching NRCs were counted again under the microscope using an eye-piece graticule. Then, an appropriate amount of virus was added into the culture medium to achieve a desirable multiplicity of infection (MOI). The adenovirus carrying HA-tagged WT telethonin or S157/161A double mutant was previously used. A MOI of 100 was recommended according to their previous study (17).

### Antibodies, western blotting, immunocytochemistry, and confocal microscopy

The rabbit nesprin antibody was generated against the synthetic polypeptide nesprin-2 (amino acids 6183-6198), as described previously (13). The dilution of nesprin-2 N3 rabbit antibody for Western blotting and immunofluorescence staining was 1:2500 and 1:250 respectively. All other antibodies including telethonin (sc-25327, Santa Cruz) and FHL-2 (K0055-3, MBL) used for Western blotting and immunofluorescence staining in this study were commercially available and diluted as recommended by the manufacturer. Western blots and immunocytochemistry staining were performed following the standard procedures described before (3, 13). The co-localization between nesprin-2 and sarcomeric markers was observed using immunostained cardiac tissue or cardiomyocytes and then analyzed in ImageJ FIJI (v1.54f).

### Immunoprecipitation (IP)

Protein lysates from neonatal rat heart tissue (homogenized using magnetic beads in the Bertin Instruments Precellys24 Homogenizer) or differentiated myotubes were harvested using IP buffer (10 mM HEPES, pH 7.4, 100 mM KCl, 5 mM EDTA, 1% Triton X-100 and protease inhibitor cocktail) and sonicated on ice. Following centrifugation at 16,000*g* for 15 min at 4 °C. The supernatant was then pre-cleared with protein-G agarose beads (Sigma). The pre-cleaned protein lysates were incubated with telethonin antibody (sc-25327, Santa Cruz), or FHL-2 (K0055-3, MBL) or mouse IgG (2 mg antibody/100 mg protein lysates) at 4 °C overnight, and then incubated with protein-G agarose beads for 2 h. After washing with IP buffer four times, the precipitated protein was washed off the beads using four times loading buffers and then subjected to Western blotting (13).

### GST pull-down assays

GST fusion nesprin proteins and GST alone were induced from 100 ml of bacterial culture for 2 h at 30 °C, with 100 μM IPTG added. GST fusion proteins were harvested and purified using glutathione-Sepharose 4B beads (Amersham Biosciences) following the manufacturer's protocol. Binding assays were performed as described before (7). 1 μg purified HA or His-telethonin recombination protein (17) was incubated with 50 μl beads for 2 h at 4 °C with rotation. Bound proteins were washed off the beads using 4 times loading buffer and then subjected to Western blotting.

### Cell culture, myoblast differentiation, and H9C2 phenylephrine stimulation

C2C12 myoblasts were cultured at 37 °C/5% CO_2_ in Dulbecco’s modified Eagle’s medium (DMEM) supplemented with 10% fetal calf serum (FCS) and 1% Pen-Strep-Glutamine (PSG). For myoblast differentiation, C2C12 were cultured in low-mitogen medium (DMEM supplemented with 1% PSG and 2% horse serum) as described previously (13), and cell lysates were collected for further investigation at day 5 to 6. Rat cardiomyoblast cells (H9C2 cells) were obtained from the ATCC and cultured in culture medium: DMEM with the addition of 10% (v/v) heat-inactivated FCS, 100 IU/ml penicillin, 100 μg/ml streptomycin, and 2 mM L-glutamine. The cells were cultured in a 5% CO2 atmosphere at 37 °C. To induce hypertrophy, H9C2 cells were starved for 18 h in DMEM containing 1% FCS and subsequently treated with maintenance medium containing DMSO (0.1% v/v), phenylephrine (PE) (Sigma) (50 μM, 100 μM) for 24 h, 48 h (29).

### Transverse aortic constriction

Minimally invasive Transverse Aortic Constriction (TAC) was performed in 10- to 12-week-old male WT (C57BL/6) mice under 1.5% isoflurane anesthesia as described previously (32). A 6/0 suture was used to constrict the aortic arch around a 27-gauge needle. Sham animals underwent a similar procedure except for aortic constriction. Experiments were performed 2 weeks after surgery.

### Statistics

Prism v9.5.1 was used for all statistical analysis. Shapiro-Wilk test and Kolmogorov-Smirnov test were used for examining the normal distribution of data. All continuous variables were presented as mean ± SD. The percentage of cells with predominant nuclear or cytoplasm/sarcomere nesprin staining and the binding between nesprin and WT/mutated telethonin and FHL-2 in IP assay were analyzed by Student’s *t* test (unpaired, two-tailed). In the GST pull-down assay, the differences of mean binding between nesprin and WT/mutated telethonin were analysed by Student’s *t* test (unpaired, two-tailed) or One-way ANOVA with Bonferroni post-hoc test. *p* values less than 0.05 were considered statistically significant.

The colocalization between nesprin-2 and sarcomeric markers was analyzed using immunostained cardiac tissue or cardiomyocytes in ImageJ FIJI (v1.54f). The JACoP Plugin was used to carry out the co-localization. Briefly, images were thresholded and then analyzed to give the Pearson’s correlation coefficient. Pearson’s correlation coefficient defines perfect co-localization as a value of 1 and no co-localization as 0. Profile plots were generated by ImageJ FIJI and graphed using GraphPad Prism (v9.5.1).

## Data availability

All data supporting the findings of this study are available within this manuscript. All the sequence data are under submissions to public databases and will be available at the date of publication.

## Supporting information

This article contains supporting information.

## Conflict of interest

The authors declare that they have no conflicts of interest with the contents of this article.
