# Supplementary material for: Nesprin-2 is a novel scaffold protein for telethonin and FHL-2 in the cardiomyocyte sarcomere
Source: J Biol Chem. 2024 Apr 2;300(5):107254. doi: 10.1016/j.jbc.2024.107254 (PMC11078644; doi:10.1016/j.jbc.2024.107254)
Supplement: Supporting Figures [file mmc1.pdf]

Supporting Information

Nesprin-2 is a novel scaffold protein for telethonin and FHL-2 in the cardiomyocyte sarcomere

Chen Li[1,2‡], Derek T. Warren[1,3‡], Can Zhou[1], Shanelle De Silva[1], Darren G.S.Wilson[1], Mitla Garcia-Maya[4], Matthew A Wheeler[5], Peter Meinke[6], Greta Sawyer[1], Elisabeth Ehler[1,4], Manfred Wehnert[7], Li Rao[2], Qiuping Zhang[1*], Catherine M. Shanahan[1*]

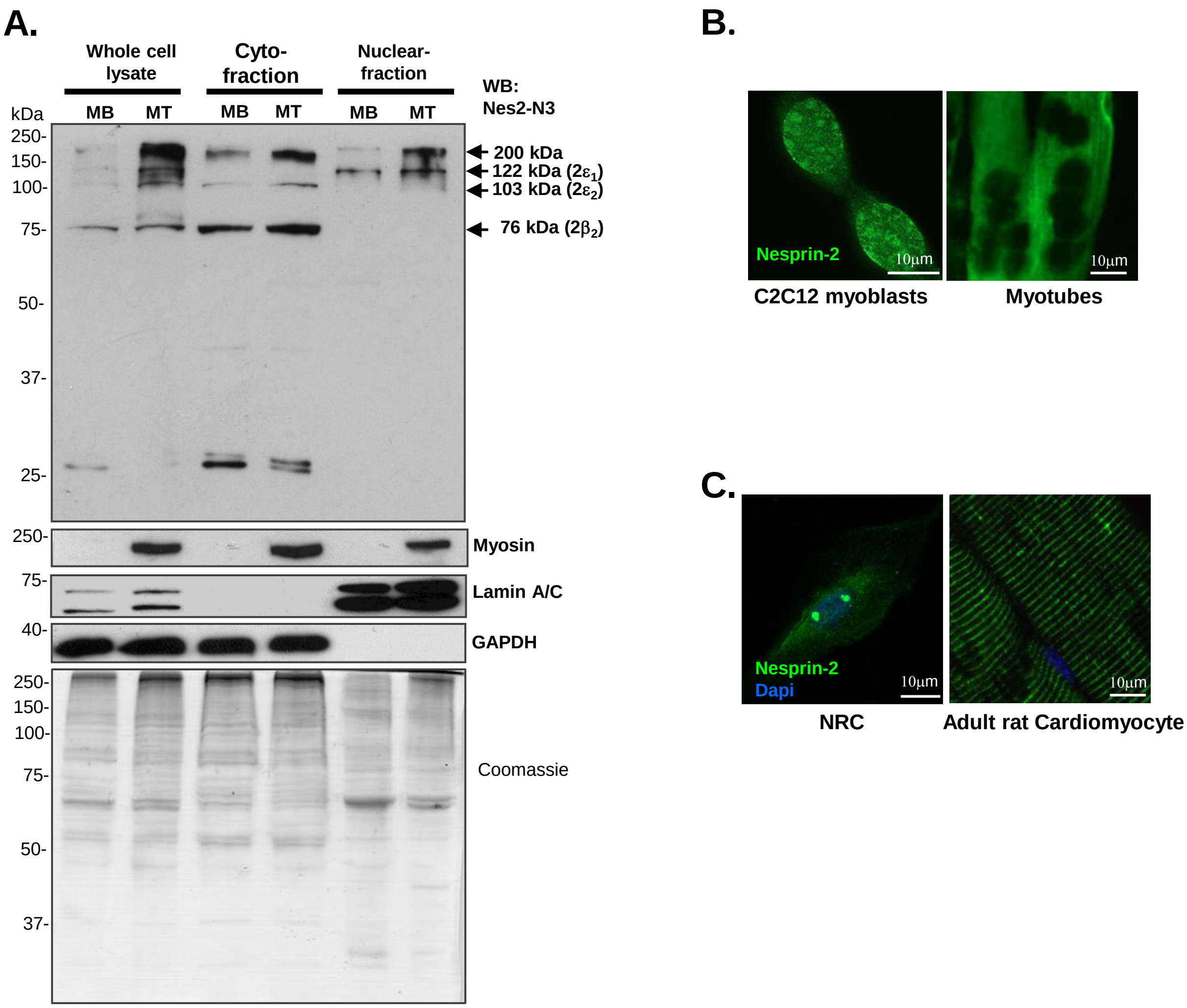

Suppl. Fig. S1

(A) Cell fractionation and Western blot demonstrated dramatic changes in production of nesprin-2 isoforms during C2C12 myoblast differentiation, (B) Immunofluorescence showed that nesprin-2 redistributed from the NE and nucleus in myoblasts (MB) to the sarcomere in myotubes (MT) during *in vitro* differentiation of mouse C2C12 myoblasts, (C) Immunofluorescence showed nesprin-2 localises to the nucleus with strong nucleolar staining in neonatal cardiomyoctyes and is present in the sarcomere in adult rat cardiomyocytes.

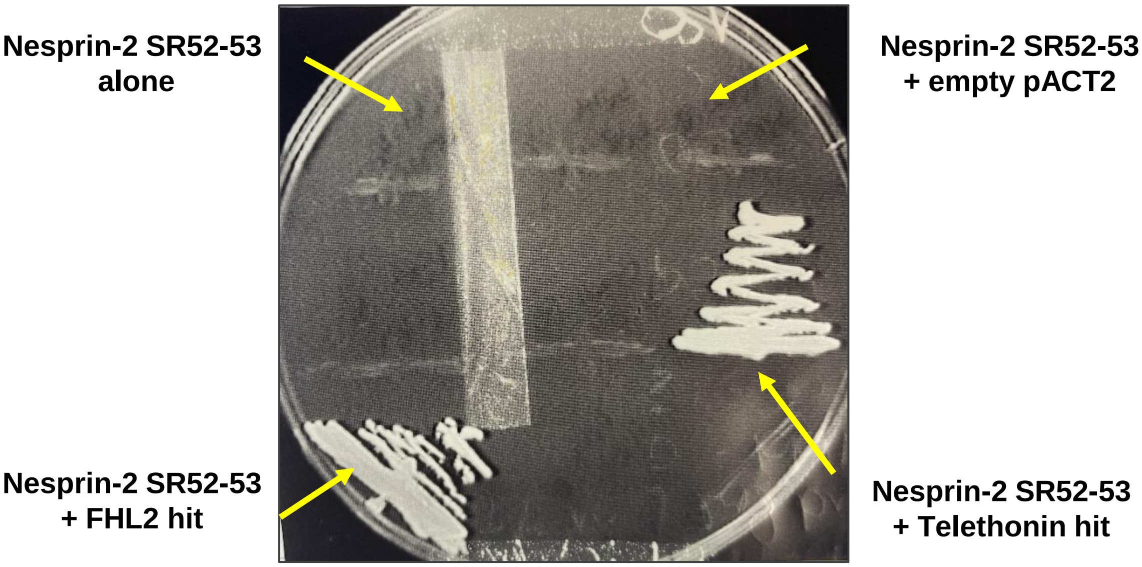

Suppl. fig. S2

Nesprin-2 SR52-53 region interacts with telethonin and FHL-2 via Yeast two-hybrid. Selection plates were streaked with yeast strain AH109 transformed with Nesprin-2 SR52-53 alone (top left), Nesprin-2 SR52-53 + empty pACT2 bait vector (top right), and colonies from the Y2H screening plates. Yeast expressing nesprin-2 SR52-53 and either FHL-2 (bottom left) or telethonin (bottom right) constructs successfully grew on the selection plates and indicated interaction.

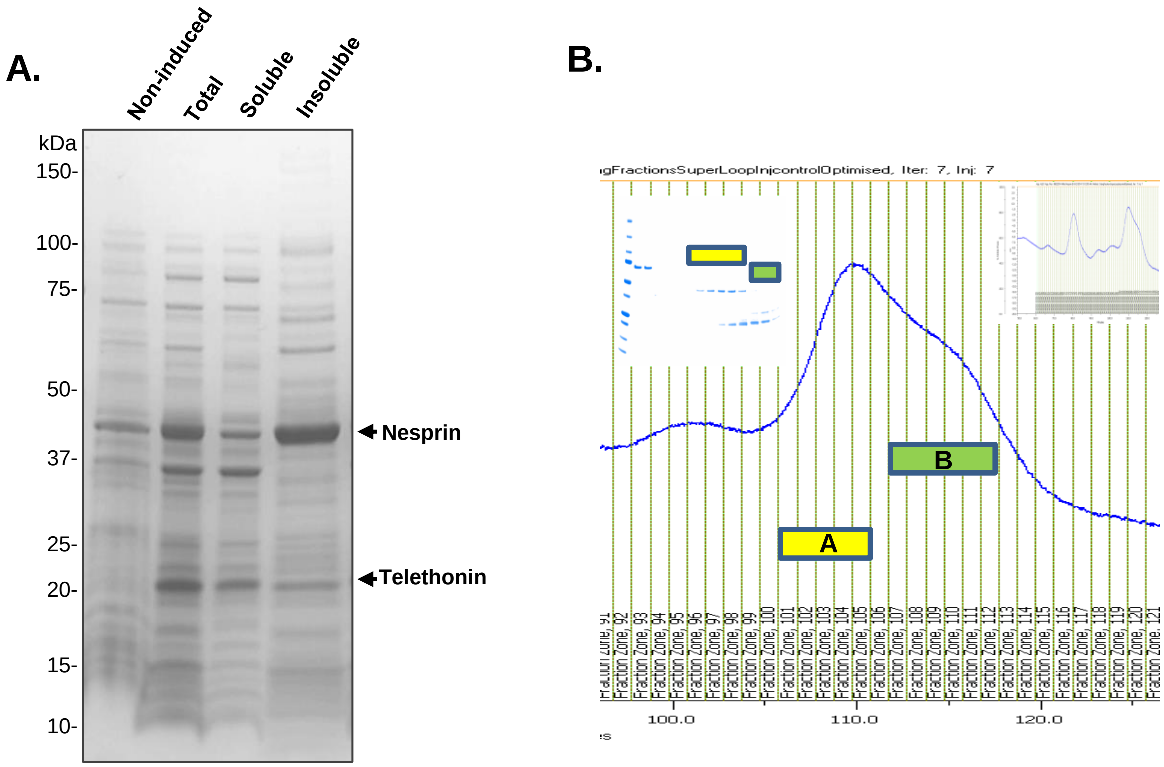

Suppl. fig. S3

(A) The plasmids containing His-telethonin and nesprin-2 SR51-53 (no tag) were co-transformed together in BL21(DE3)pLysS *E.coli* strain. Expression was done in LB with chloramphenicol, ampicillin and streptomicin until OD600 was 0.5 and induced by 0.4mM IPTG and overnight incubation at 18ºC at 200rpm. Coomassie staining showed total, soluble and insoluble fractions of bacterially expressed and purified His-telethonin and nesprin-2 SR51-53. Nesprin-2 is predominantly insoluble but a significant proportion of telethonin is soluble (arrowed). For further size exclusion chromatography (SEC), only the soluble fractions were used.

(B) SEC chromatogram. Fractions from Nikel affinity column eluted with 600mM imidazole (data not showed) were pooled and loaded onto a Superdex 200 16/60 column equilibrated with 50mM Tris/HCl, 150mM NaCl, 20mM βME (right insert, full chromatogram). 2ml fractions were collected at a flow rate of 2ml/min, aliquots were taken and run into an 8-16% SDS-PAGE stained with Coomassie blue (left insert). Yellow box A indicates nesprin-2 elution and green box B indicates telethonin elution. The fractions 101-112 were pooled and loaded on to a Superdex 75 10/300 column to examine if the complex could be separated from free telethonin. 100mM βME was added to this equilibration/elution buffer to reduce aggregation.

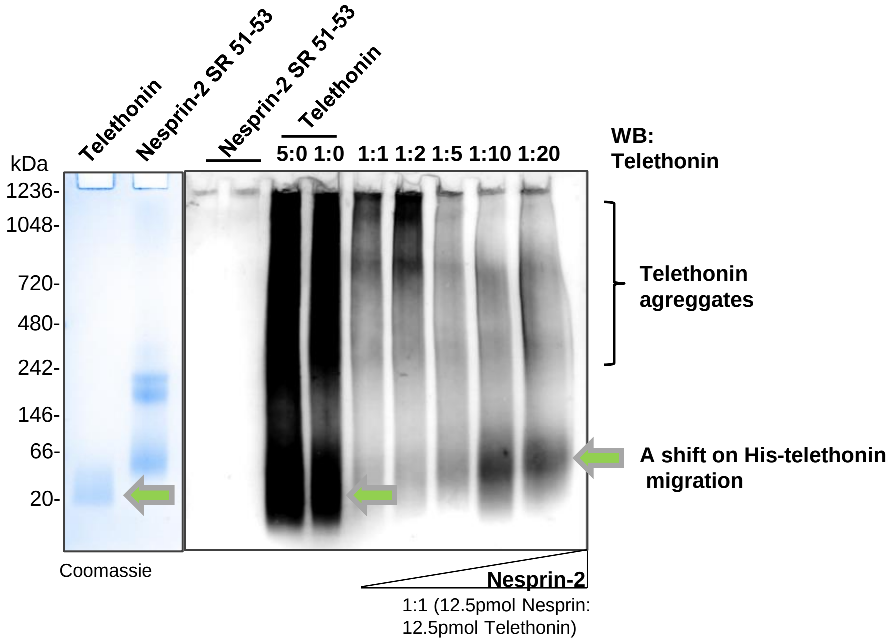

Suppl. Fig. S4

Nesprin2-SR 51-53 and telethonin in a NOVEX native gel (4-16%). The gel on the left shows Coomassie staining of His-telethonin and His-nesprin on their own. Both proteins run as monomers, but also formed aggerates. Western blot on the right shows the interaction between telethonin and nesprin-2 as a band shift migration. The blot shows nesprin alone was not detected by telethonin antibody (sc-25327, Santa Cruz, the first two lanes on the right panel). The other lanes show telethonin on its own forms several aggregates (compare 1:0 ratio vs 1:10), with presence of increasing nesprin-2 SR51-53 concentrations, almost all aggregates disappeared as shown by arrows, suggesting that nesprin-2 has some effect on telethonin behaviour with their interactions.

***FHL-2* gene: Exon 3, c.337 C>T p.R113C**

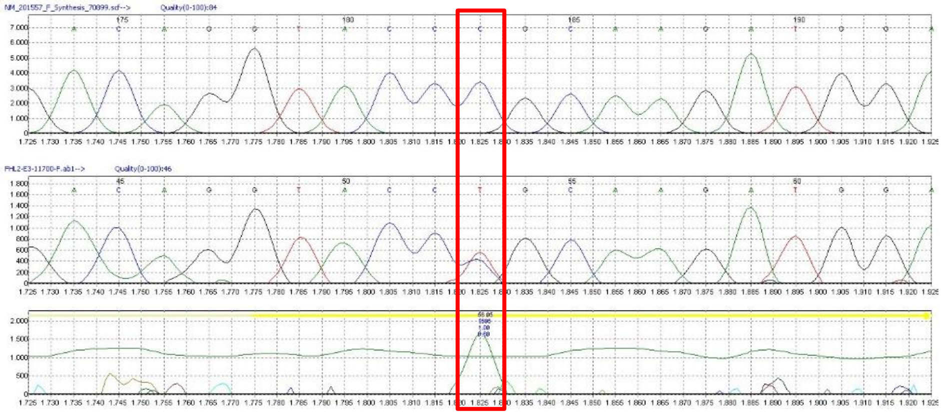

Suppl. fig. S5

A novel missense mutation R113C of FHL2 (c.337 C>T, p.R113C, rs140148322) was identified in the LIM2 domain of the *FHL-2* gene in an EDMD with CD patient. PolyPhen-2 report for R113C predicted this mutant probably damaging.
